# Microencapsulation of rice bran oil using pea protein and maltodextrin mixtures as wall material

**DOI:** 10.1016/j.heliyon.2020.e03615

**Published:** 2020-04-01

**Authors:** Ó. Benito-Román, T. Sanz, S. Beltrán

**Affiliations:** Department of Biotechnology and Food Science (Chemical Engineering Section), Faculty of Sciences, University of Burgos, Plaza Misael Bañuelos s/n, 09001 Burgos, Spain

**Keywords:** Food science, Food technology, Pea protein, Encapsulation, Microfluidization, Rice bran oil, Spray drying, PGSS

## Abstract

In this work, the encapsulation of rice bran oil extracted using supercritical CO_2_ has been studied. In the first stage, the emulsification process by high pressure homogenization was studied and optimized. The effect of the working pressure (60–150 MPa), the composition of the carrier (mixtures of pea protein isolate (PPI) and maltodextrin (MD), from 50 to 90% of PPI) and the carrier to oil ratio (2–4) on the emulsion droplet size (EDS) was studied. To minimize the EDS, moderate pressures (114 MPa), a carrier composed mainly by PPI (64%) and carrier to oil ratios around 3.2 were required. The emulsion obtained in the optimal conditions (EDS = 189 ± 3nm) was dried using different technologies (spray-drying, PGSS-drying and freeze drying). The supercritical CO_2_ based drying process (PGSS) provided spherical particles that resulted in the smallest average size (but broader distribution) and lower encapsulation efficiency (53 ± 2%).

## 1 Introduction

Rice bran oil is a source of bioactive molecules such as sterols, tocols, γ-oryzanols and unsaturated fatty acids (Friedman, 2013; Gul et al., 2015), which makes it an excellent candidate to be used to enrich foods. In order to avoid the change of the organoleptic properties in the products where incorporated, as a consequence of the oxidative degradation of the oil (Bakry et al., 2016), these molecules must be protected and stabilized. Microencapsulation, known as the technique in which drops of a core bioactive material are surrounded by a coating agent (Gallardo et al., 2013), seems to be a suitable approach to protect rice bran oil. By this process, the oil is converted in a fine dry powder that can be easily handled and used as additive in different matrices (Hee et al., 2015). The microencapsulation processes of oils are sequential and involve the formation of an emulsion (Jafari et al., 2007b) that will be subsequently dried to form microparticles.

Emulsification methods can be divided in low and high energy methods, such as microfluidization or high pressure homogenization (Zhang et al., 2015). This method can be used at industrial scale due to its flexibility to control the emulsion droplet size (EDS) and the ability to produce emulsions from a variety of materials (Jafari et al., 2007a). Microfluidization processes are complex since the emulsification process is affected by several parameters that usually present important interactions: pressure and number of homogenization cycles (together determine the energy input), the carrier material, the carrier to core ratio, and the solids content in the emulsion (Sadeghpour Galooyak and Dabir, 2015). Therefore, a careful control of the process parameters are key to ensure the stability of the emulsion, since there is a correlation between stability and encapsulation efficiency (Carneiro et al., 2013).

Polysaccharides are the most extensively used wall materials to encapsulate oils. However, vegetable proteins are a new generation of wall materials, trendy in encapsulation processes in pharma, cosmetics and food industries (Gumus et al., 2017) because of the advantages they offer (Nesterenko et al., 2013). Among the different vegetable proteins available, pea proteins are those that present the most interesting properties: emulsifying properties, high nutritional value, functional properties, nonallergenic characteristics (Moreno et al., 2016), and wall forming properties for microencapsulation (De Graaf et al., 2001). In fact, it is possible to find in the literature a few examples of researchers that use pea proteins to encapsulate bioactive compounds, such as grape marc extract (Moreno et al., 2016), ascorbic acid (Pereira et al., 2009; Pierucci et al., 2006), α-tocopherol (Pierucci et al., 2007), triglycerides (Gharsallaoui et al., 2010), or lycopene (Ho et al., 2017). Pea protein isolates (PPI) present disadvantages related to pH sensitivity and high viscosity solutions at high concentrations, since PPI are complex mixture of proteins (Nesterenko et al., 2013). The combination of pea proteins with polysaccharides seems to be able to overcome some of the limitations that pea proteins present when used alone (Dickinson, 2003). In general, the use of combinations of proteins and polysaccharides produces an increase of the emulsion stability among other functional properties (Benichou et al., 2002).

Once the emulsion is prepared, it is necessary to convert it into dry solid particles in which the core is the valuable material to be preserved. Spray drying has been the conventional technique to obtain dry particles from emulsions, due to several advantages it offers, such as simplicity or continuous operation (Jafari et al., 2008a). Freeze drying is an interesting alternative since it works at low temperatures and vacuum conditions in order to sublime the water. Its main drawback is the energy consumption and long residence times that result in high processing costs, which can be up to 30–50 times higher than spray-drying (Calvo et al., 2010). An alternative particle formation technology is the PGSS (Particles from Gas Saturated Solutions) drying, which is based on the use of supercritical carbon dioxide (SC-CO_2_) as drying agent. In this technique, an aqueous solution saturated with SC-CO_2_ is expanded through a nozzle. This sudden depressurization promotes the rapid vaporization of the gas dissolved (enhancing atomization), that together with the intense cooling due to the Joule-Thomson effect that CO_2_ expansion promotes (Martín and Weidner, 2010), enhance particle formation. The advantages of this technique are the mild temperatures achieved in the drying process (40–80 °C) (De Paz et al., 2012) as well as the oxygen free atmosphere. These are excellent properties to preserve the activity of the bioactive compounds present in the rice bran oil.

The aim of this work is to study the microencapsulation process of rice bran oil using mixtures of pea protein and maltodextrin as wall material: first, using response surface methodology to study and optimize the emulsion formation process and second, studying the effect of the drying method on the properties of the formed particles.

## 2 Materials and methods

### 2.1 Rice bran oil

Supercritical CO_2_ extracted rice bran oil was purchased at All Organic Treasures GmbH (Wiggensbach, Germany). It was centrifuged (45 min at 25 °C and 4500 rpm) in order to remove a heavy fraction of waxes. A complete characterization (including total phenolics, total flavonoids, γ-oryzanol, total tocopherol, fatty acid profile and antioxidant activity using the DPPH radical) was carried out, following the analytical procedures described in Benito-Roman et al. (Benito-Román et al., 2019), briefly summarized in section 2.1.1.

#### 2.1.1 Rice bran oil characterization: analytical methods

##### 2.1.1.1 Total phenolics content (TPC)

The total phenolics content was measured using the Folin-Ciocalteu method: 0.5 mL of the oil dissolved in ethanol (5 mg/mL) were mixed with 0.5 mL of the Folin-Ciocalteu reagent, 5 mL of distilled water and 1 mL of sodium carbonate solution (20%). The mixture, that was vigorously stirred and let in darkness for 1 h at room temperature, was subsequently centrifuged (13300 rpm for 10 min) and filtered (0.45 μm, pore size) in order to get a completely clear liquid that was measured at 750 nm using the V-750 (Jasco Corporation, Tokyo, Japan) spectrophotometer. As standard curve, different concentration gallic acid solutions prepared in ethanol were used, and the TPC was expressed as mg Gallic Acid Equivalent/g of extracted oil.

##### 2.1.1.2 Total flavonoids content (TFC)

In this procedure 0.5 mL of the oil solution (5 mg/mL in ethanol) were mixed with 2 mL of distilled water and 0.15 mL of NaNO_2_ solution (5%, w/v). Then, 6 min later, 0.15 mL of AlCl_3_ (10%, w/v) were added. 6 min later, 1 mL of NaOH (1M) and 1.2 mL of ethanol were added. All this procedure was carried out at room temperature. The absorbance of the mixture, previously filtered (0.45 μm), was measured at 415 nm spectrophotometer V-750, (Jasco Corporation, Tokyo, Japan). A quercetin standard curve prepared in ethanol was used to calculate the TFC of the samples (expressed as mg Quercetin Equivalent/g of extracted oil).

##### 2.1.1.3 Determination and quantification of the γ-oryzanol profile

γ-oryzanols were determined by HPLC (Series 1100, Agilent, Santa Clara CA, USA) using a DAD detector (330 nm). Accurately weighted oil samples (approximately 30 mg) were dissolved in 1 mL of 2-propanol and filtered (0.45 μm pore size). 10 μL of oil solution were injected in a Zorbax XDB C18 5 μm 150 mm × 4.6 mm column (Agilent, Santa Clara CA, USA). The mobile phase used consisted of acetonitrile (Merck KGaA, Darmstadt, Germany), methanol (HiPersolv Chromanorm, VWR, USA) and 2-propanol (Merck KGaA, Darmstadt, Germany) in 50/40/10 proportion. 1-Cycloartenylferulate, 2-24-Methylene-cyclo-artanylferulate, 3-Campesterylferulate, 4-β-Sitosterylferulate were the γ-oryzanol standards used. The results were expressed as the sum of each individual component in order to calculate the total mg of γ-oryzanol/g of extracted oil.

##### 2.1.1.4 Determination and quantification of the tocopherol profile

Tocopherols determination was done by HPLC-DAD after a solid phase extraction (SPE) stage, according to the procedure described by Rebolleda et al. (Rebolleda et al., 2012). Silica cartridges (1000 mg/6 mL, Sep-Pak®, Waters Corporation, Milford MA, USA) were initially prepared by adding 5 mL of n-hexane before the addition of 1 mL of the oil solution (previously dissolved in hexane in order to have a concentration equal to 0.1 g/mL). Then the tocopherols were eluted in a three steps procedure: first 5 mL of n-hexane were passed through the cartridge, then 5 mL of n-hexane:diethyleter (99:1, v/v) were passed (and discarded) and finally 50 mL of n-hexane:diethyleter (99:2, v/v) were used. The very last fraction was collected and evaporated at temperatures lower than 40 °C, under vacuum conditions. The solid residue obtained in the flask was weighted and re-dissolved in 1.5 mL of n-hexane. 50 μL of this solution were injected in the HPLC system described next: model Agilent series 1100, Santa Clara CA, USA, equipped with a ACE 5 Silica (250 × 4.6 mm) column, using as mobile phase a solution of 99% hexane (A) and 1% 2-propanol (B) at a flow rate of 1 mL/min, operated in isocratic mode. A diode array detector was used at 296 nm.

##### 2.1.1.5 Antioxidant activity assay

The antioxidant activity (AA) of the rice bran oil was evaluated using the ABTS^·+^ radical scavenging assay, following the procedure described by Benito-Román et al. (2019). The commercial oil was dissolved in ethanol in order to get solutions in the range of concentrations from 0.25 to 5 mg/mL. 3 mL of the already prepared ABTS^·+^ were added to 1 mL of each oil solution. The absorbance was measured at 734 nm (spectrophotometer V-750, (Jasco Corporation, Tokyo, Japan)) after being incubated at room temperature for one hour. The inhibition percentage calculated for each oil solution versus oil concentration was represented. Then, the concentration of oil required for a 50% inhibition of the radical ABTS^·+^ was calculated and expressed as IC_50_ value. The interpretation of the results indicates that the lower the concentration of oil needed to inhibit the activity of the ABTS^·+^ radical by 50%, the higher the antioxidant activity of the sample.

##### 2.1.1.6 Determination and quantification of the fatty acids profile

The fatty acids profile was determined by the AOAC official method (AOAC, 1995) by gas chromatography using a 6890N Network GC System (Hewlett Packard, Palo Alto CA, USA) equipped with an auto-sampler (model 7683B) and a flame ionization detector (FID). Hellium was used as carrier at a flow rate of 1.8 mL/min. A fused silica capillary column (OmegawaxTM-320, 30 m × 0.32 mm; Sigma Aldrich Co., USA) was used. The quantification of the major fatty acids was made by relating the peaks area to the area of an internal standard (methyl tricosanoate) as indicated by the AOAC method (AOAC, 1995).

### 2.2 Microemulsions preparation

In this work the O/W emulsions were prepared to have a 15% content in solids, using water as continuous phase and rice bran oil as dispersed phase. Different wall materials (carriers) were tried, consisting in mixtures of pea protein isolate Pisane C9 (Cosucra (Warcoing, Belgium) was kindly provided by InnovaFood (Barcelona, Spain)) and maltodextrin (MD, purchased at Myprotein (Northwich, United Kingdom)). The PPI content in the mixture ranged from 50% to 90%.

The selected wall materials were dissolved in distilled water at 30 °C. A coarse emulsion was prepared by addition of the rice bran oil. The mixture was stirred for 10 min and a coarse emulsion was formed after being stirred in a high speed stirrer for 3 min at 21.000 rpm. The resulting coarse emulsion was then passed through the microfluidizer LM-20 (Microfluidics, Westwood, MA, USA) provided with the interaction chamber F20Y at different pressures and up to 8 passes. A cooling coil immersed in ice water was used to keep the emulsion at controlled temperature throughout the homogenization process. O/W emulsions were produced in batches of 150 g.

#### 2.2.1 Experimental design used for the emulsion preparation

The optimization of the emulsification process was done using the surface response methodology, following a face centered central composite design. A three factor, three level design including five repetitions of the center point was employed. The selected variables were homogenization pressure (60–150 MPa), percentage of PPI in the carrier material (from 50% to 90%), and carrier to oil ratio (COR), which ranged from 2 to 4. The response variable studied was the emulsion droplet diameter, and the goal was to minimize it. The experimental data were fitted to a quadratic model. The analysis of variance (ANOVA) was conducted to determine the significance of the model (determination of the regression coefficient R^2^) and the statistical effect of the process parameters on the response surface, as well as the interaction between the experimental factors. A 95% confidence level was used in all the cases, and the statistical analysis of the experimental results was done using the Statgraphics X64 software.

#### 2.2.2 Characterization of the O/W emulsions

##### 2.2.2.1 Droplet size characterization

The droplet size distribution of the O/W emulsions was measured using the Mastersizer 2000 (Malvern Panalytical, Malvern, United Kingdom) device. Around 1 mL of the emulsion was suspended in distilled water, at stirring rate around 1000 rpm. Each emulsion droplet size was measured three times. Droplet sizes were calculated according to Eqs. [1], [2]; where equation [1] refers to the mean diameter over volume (DeBroukere mean, D[4,3]) and equation [2] refers to the volume/surface mean diameter (Sauter mean, D[3,2]):

(1) $$D\left[4,3\right]=\frac{\sum_{1}^{n}D_{iv_{i}}^{4}}{\sum_{1}^{n}D_{iv_{i}}^{3}}\;\;\;\;\;$$

(2) $$D\left[3,2\right]=\frac{\sum_{1}^{n}D_{iv_{i}}^{3}}{\sum_{1}^{n}D_{iv_{i}}^{2}}\;\;\;\;\;$$

The span value was also calculated according to Eq. [3], where d_0.5_ refers to the median particle size; whereas d_0.1_ and d_0.9_ refer to the maximum particle diameter below which 10% and 90% of the sample volume exists, respectively.

(3) $$span=\frac{d_{0.9}-d_{0.1}}{d_{0.5}}\;\;\;\;\;$$

When the average particle size distribution of solid particles was measured, absolute ethanol (VWR Chemicals, USA) was used to disperse them, in order to avoid the re-dissolution in water.

##### 2.2.2.2 Emulsions stability

Physical stability of the O/W emulsion was analyzed by static multiple scattering in a vertical scan analyzer Turbiscan Lab Expert (Formulaction, Toulouse, France) with ageing station AGS. The stability of the original emulsion at 25 °C was monitored at 3 h intervals for 7 days.

##### 2.2.2.3 ζ-potential measurement

The ζ-potential measurement of the optimal emulsion was conducted using the Zetasizer Nano ZS apparatus (Malvern Panalytical, Malvern, United Kingdom), using the Laser Doppler Velocimetry technique, using the DTS1061 disposable folded capillary cell. Five replicates of 12 measurements were performed for each sample at 20 °C. The results presented in this work show average values of the 6 runs with the relative measurement error.

### 2.3 Microemulsions drying

#### 2.3.1 Spray drying

The spray-drying process was carried out in a commercial B-290 mini Spray-dryer (BÜCHI Labortechnik AG, Flawil, Switzerland). The O/W emulsion, obtained as described in Section 2.3, was fed into the spray-drying apparatus at 3.0 g/min. It was sprayed through a nozzle with 1.5 mm diameter and dried under a N_2_ flow of 360 L/h set at 155 °C. Outlet temperature was kept in the range from 92 to 96 °C.

#### 2.3.2 PGSS drying

The PGSS drying apparatus was extensively described by Melgosa et al. (Melgosa et al., 2019). The O/W emulsion was pumped into the static mixer by a GILSON 305 piston pump; whereas CO_2_ was pumped in order to have a gas to product ratio (GPR) equal to 30 g/g. Both CO_2_ and O/W emulsion were mixed in a static mixer (105 °C, pressure 10 MPa). The gas-saturated emulsion was then expanded into the spraying tower through a capillary nozzle with an internal diameter of 400 μm (Spraying Systems, model PF1650-SS). The spraying tower was made of PVC and was heated at 55 °C. CO_2_ was vented off the spraying tower and passed through a water vapor condenser before final release. After all, the emulsion was pumped, and CO_2_ was allowed to flow through the system at the same pressure and temperature conditions during 15 min in order to completely dry the particles. After that, the system was depressurized and particles were collected from the walls and bottom of the spraying tower and stored in darkness and refrigeration at 4 °C for subsequent analyses.

Pressure in the static mixer and temperatures in the static mixer and the spraying tower were selected based on previous studies (Melgosa et al., 2019).

#### 2.3.3 Freeze drying

The emulsion was placed in a Petri dish (15 cm diameter) forming a layer of 1 cm. It was equilibrated at -80 °C for 2 h and freeze-dried in a Labconco Freeze Dry System (Labconco Inc., Kansas City MO, USA) at 0.15 mbar for, at least, 48 h. Once the emulsion was completely dried, it was carefully crushed in order to obtain fine powder.

#### 2.3.4 Dry powders characterization

##### 2.3.4.1 Moisture

Moisture content of the dried particles was determined gravimetrically. Samples (approximately 0.5 g) were weighed before and after drying in an oven at 105 °C for 24 h.

##### 2.3.4.2 Encapsulation efficiency

Encapsulation efficiency (EE) was calculated according to the equation [4], where TO stands for total oil and SO for surface oil. SO refers to the fraction of oil that can be easily extracted with organic solvents without disruption of the solid matrix (Gallardo et al., 2013).

(4) $$EE\;\left(\%\right)=\left(\frac{TO-SO}{TO}\right)\times 100$$

For the **non-encapsulated oil** determination, 0.25 g of solid particles were suspended with 15 mL of hexane in a Falcon centrifuge tube, which was vortexed for 15 s at room temperature and centrifuged at 3000 rpm during 20 min. Immediately afterwards, the supernatant was taken and filtered (0.45 μm), and its oil content was measured spectrophotometrically at λ = 314 nm. A calibration curve was previously constructed using SC-CO_2_ extracted rice bran oil dissolved in hexane. **Total oil** was determined according to the procedure presented by Blich and Dyer (Blich and Dyer, 1959) with some modifications. In brief, 0.25 g of solid particles were weighted and suspended in 3.8 mL of a mixture chloroform/methanol/water (in proportions 1/2/0.8). Mixture was vigorously stirred in a vortex mixer, and the centrifuged (2 min at 5000 rpm). Then, 2 mL of chloroform, 2 mL of methanol and 1.8 mL of water were added to the sample, which was again stirred in the vortex mixer and centrifuged (2 min at 5000 rpm). After that, the oily phase was carefully extracted with a needle and transferred to a vial. It was allowed to stay at room temperature for long enough so all the chloroform could be removed from the sample. The oil was determined gravimetrically.

##### 2.3.4.3 Particles morphology

Morphology of the dried particles was observed by Scanning Electron Microscopy (SEM) using the microscope Quanta 600 (Thermo Fisher Scientific, Walthman MA, USA) operated at 20 kV under high vacuum conditions. Samples were gold-sputtered and observed with magnifications of 500, 1000 and 2000× for PGSS and spray-dried particles, and 40, 100 and 400 × for the freeze-dried powders.

## 3 Results and discussion

### 3.1 Characterization of the rice bran oil

In Table 1 the main features regarding the composition of the rice bran oil used in this work are presented.

**Table 1:** Composition of the rice bran oil used in this work.

Parameter	Result
Total Phenolics (mg GAE/g oil)	2.1 ± 0.1
Total Flavonoids (mg QE/g oil)	2.4 ± 0.2
Antioxidant Activity – IC_50_ (mg/mL)	0.6 ± 0.1
Total Tocopherols (mg/g oil)	2.24 ± 0.05
Total γ-oryzanol (mg/g oil)	16.6 ± 0.1
Total Fatty Acids (mg/g oil)	960 ± 5

Rice bran oil presents a wide profile of bioactive molecules. More specifically, α-tocopherol accounted for the 92% of the total tocopherols detected (β and γ, accounted for 3 and 5%, respectively), whereas the individual components of γ-oryzanol profile presented the following order of prevalence: 24-Methylene cycloartanyl ferulate (7.12 ± 0.03 mg/g of oil) > Cycloartenyl ferulate (4.88 ± 0.01 mg/g of oil) > Campesteryl ferulate (2.91 ± 0.12 mg/g of oil) > β-sitosteryl ferulate (1.71 ± 0.01 mg/g of oil). The fatty acid (FA) profile was composed by monounsaturated FA (40.6%), polyunsaturated FA (40.4%) and saturated FA (19.0%). Jesus et al. (Jesus et al., 2010) reported a rice bran oil profile composed mainly by monounsaturated FA (42.8%), polyunsaturated FA (31.4%) and saturated FA (25.8%), proportions slightly different from those obtained for the commercial rice bran oil used in this work. The main fatty acids detected were oleic (370 ± 2 mg/g oil), linoleic (374 ± 1 mg/g oil) and palmitic (156 ± 1 mg/g oil), similar to those results presented by Balachandran et al. (2008), who reported oleic, linoleic and palmitic, as the major fatty acids extracted from rice bran when using SC-CO_2_.

Benito-Román et al. (2019) studied the extraction of rice bran oil using supercritical CO_2_ in a pressure range from 30 to 40 MPa and in a temperature range from 40 to 60 °C. The commercial oil used in this work has similar antioxidant activity to that extracted by Benito-Román el at. (Benito-Román et al., 2019), although it presents higher phenolics and flavonoids content. Benito-Roman et al. also reported total γ-oryzanol content in the range 14.9–17.6 mg/g oil and total fatty acids content in the range 818–907 mg/g oil. Other authors, such as Tomita et al. (2014) found the highest γ-oryzanol content in the oil at 40 MPa and 60 °C (12.6 mg/g of oil), whereas Yoon et al. (2014) reported concentrations of γ-oryzanols in rice bran oil in the range 19.3–31.0 mg/g of oil when working at 27.6 MPa - 60 °C and 41.4 MPa - 60 °C, respectively. Values presented in Table 1 for the commercial rice bran oil used in this work are in the same order of magnitude. As it is possible to see, it is difficult to compare the results since it is not possible to know under which conditions the commercial oil was extracted, but as demonstrated Benito-Roman et al., the supercritical CO_2_ extraction provides rice bran oil that results to be in higher quality (in terms of antioxidant activity and bioactive molecules content) than oils extracted using organic solvents, such as hexane (Benito-Román et al., 2019).

### 3.2 Selection of the number of passes through the microfluidizer

The effect of the number of passes through the microfluidizer (pressure 105 MPa) on the droplet size of an emulsion prepared using a mixture PPI/MD (70/30) with a COR equal to 3 was studied. Up to 8 passes through the microfluidizer interaction chamber were carried out. As it is presented in Table 2, with the number of passes, the average droplet size tends to decrease, with no significant reduction when moving from pass 7 (D[4,3] 250 ± 9 nm) to pass 8 (D[4,3] 229 ± 3 nm), and small reduction in the span (from 1.7 to 1.5, passes 7 and 8, respectively). However, an important decrease in droplet size was observed when moving from pass 5 (D[4,3] 378 ± 10 nm) to pass 7. After the third time the emulsion was passed through the interaction chamber, there were no statistically significant differences in the D[3,2] of the emulsion formed.

**Table 2:** Evolution of the EDS with the number of passes through the microfluidizer.

Number of passes	D[4,3] (nm)	D[3,2] (nm)	Span
Coarse Emulsion	9130 ± 193^a^	5144 ± 304^a^	2.8 ± 0.4^a^
1	1220 ± 68^b^	137 ± 1^b^	29 ± 3^b^
3	725 ± 112^c^	120 ± 3^b^	6 ± 2.^c^
5	378 ± 10^d^	113 ± 1^b^	2.08 ± 0.02^d^
7	250 ± 9^e^	110 ± 1^b^	1.72 ± 0.02^e^
8	229 ± 3^e^	111 ± 3^b^	1.52 ± 0.08^e^

∗Different letters in each column mean statistically significant differences at 95% confidence level.

**Figure 1:**
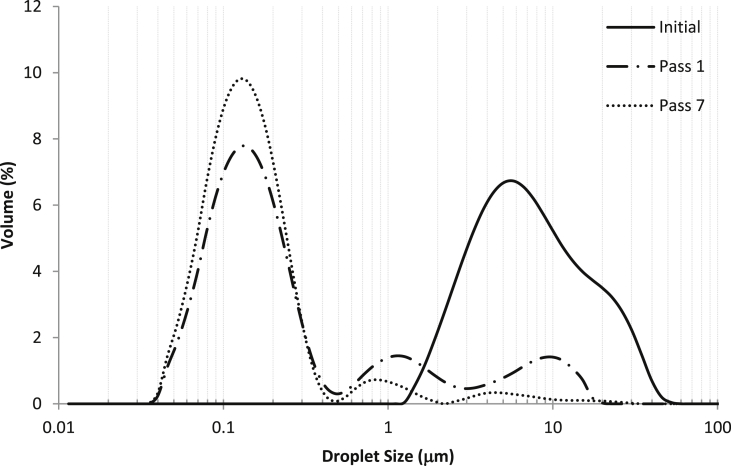
Evolution of the EDS with the number of passes through the microfluidizer (105 MPa), carrier contained 70% of PPI and the COR was 3. Initial refers to the coarse emulsion.

In Figure 1 it is possible to see how the initial monomodal but broad droplet distribution (centred in 12 μm) for the coarse emulsion evolves to a multimodal distribution with two small peaks after a main peak centred in 120 nm after 7 passes through the homogenization chamber.

The emulsion formation by high pressure homogenization is described as a two-step process (Jafari et al., 2008b): the deformation and disruption of the droplets (an increase of the specific surface area of the emulsion) is followed by the stabilization of this new surface by the action of an emulsifier (which, in order to prevent the recoalescence of the new droplets, has to adsorb on the newly created surface). Thus, the final performance of the emulsification process will depend on the equilibrium of these two competitive steps (the formation of the droplets and their stabilization). The stabilization due to the action of the emulsifier is controlled by its adsorption rate. If it is too slow, the probability of coalescence increases. In the specific case of the high pressure homogenization, a decrease in the EDS is expected when increasing the energy input (pressure and number of passes through the interaction chamber), but it is also necessary the presence of enough emulsifier to cover and stabilize the new formed droplets (Jafari et al., 2007b). Therefore, according to the results presented in Figure 1 and Table 2, the number of passes though the microfluidizer was set in 7, as increasing the number of passes did not involve a reduction in the average droplet size, and in some cases could lead to overprocessing of the sample.

### 3.3 Effect of the process parameters on the emulsification by microfluidization

In Table 3, the experimental plan and the experimental results are presented, and in Table 4, the results for the analysis of variance (ANOVA). The statistical analysis of the results reveals good agreement between the experimental results and those predicted by the model (R^2^ = 0.979). From those results it is possible to see the complexity of the studied process, since there are two process parameters (pressure and carrier to oil ratio (COR)) that have statistical effect on the response variable and moreover, there are strong interactions between some of those experimental factors (pressure and protein content; pressure and COR; and protein content and COR).

**Table 3:** Experimental plan and experimental results after 7 cycles of homogenization in the microfluidizer.

run	P (MPa)	PPI (%)	COR (-)	D[4,3] (nm)	D[3,2] (nm)	Span
1	60	50	2	1215 ± 32	145.0 ± 0.1	3.91 ± 0.09
2	150	50	2	575 ± 27	115.0 ± 0.1	1.2 ± 0.1
3	60	90	2	1178 ± 104	139.0 ± 0.6	3.2 ± 0.5
4	150	90	2	343 ± 35	111.3 ± 0.6	1.85 ± 0.03
5	60	50	4	453 ± 57	114.3 ± 0.5	2.3 ± 0.2
6	150	50	4	222 ± 12	109.3 ± 2.3	1.26 ± 0.06
7	60	90	4	860 ± 65	118.2 ± 0.1	6.0 ± 0.2
8	150	90	4	212 ± 19	109.3 ± 0.5	1.58 ± 0.04
9	60	70	3	654 ± 6	123.0 ± 0.2	2.6 ± 0.1
10	150	70	3	227 ± 10	106.7 ± 2.3	1.6 ± 0.1
11	105	50	3	210 ± 2	112.7 ± 0.6	1.12 ± 0.05
12	105	90	3	252 ± 6	110.0 ± 0.3	1.69 ± 0.01
13	105	70	2	375 ± 18	116.7 ± 0.6	1.15 ± 0.01
14	105	70	4	297 ± 9	109.0 ± 0.1	1.78 ± 0.04
15	105	70	3	268 ± 12	111.0 ± 2.0	2.19 ± 0.09
16	105	70	3	255 ± 4	110.8 ± 0.1	1.73 ± 0.01
17	105	70	3	279 ± 15	112.1 ± 0.2	1.81 ± 0.01
18	105	70	3	250 ± 9	110.3 ± 0.6	1.72 ± 0.02

**Table 4:** ANOVA table.

	Source	Sum of Squares	Df	Mean Square	F-Ratio	P-Value∗
Effect of factors	A:Pressure	763417	1	763417	159.70	0.0000
	B:Protein	4580	1	4580	0.96	0.3564
	C:COR	289000	1	289000	60.46	0.0001
Cuadratic terms of factors	A∗A	178806	1	178806	37.41	0.0003
	B∗B	3203	1	3203	0.67	0.4367
	C∗C	31829	1	31829	6.66	0.0326
Interactions between factors	A∗B	40328	1	40328	8.44	0.0198
	A∗C	51200	1	51200	10.71	0.0113
	B∗C	63013	1	63013	13.18	0.0067
	Total error	38242	8	4780		
	Total (corr.)	1.798E6	17			

∗ Critical p-value at a 95% confidence level is 0.05. p-values below the critical value indicate the statistical significance of the factor studied.

#### 3.3.1 Single effect of the process parameters

**Figure 2:**
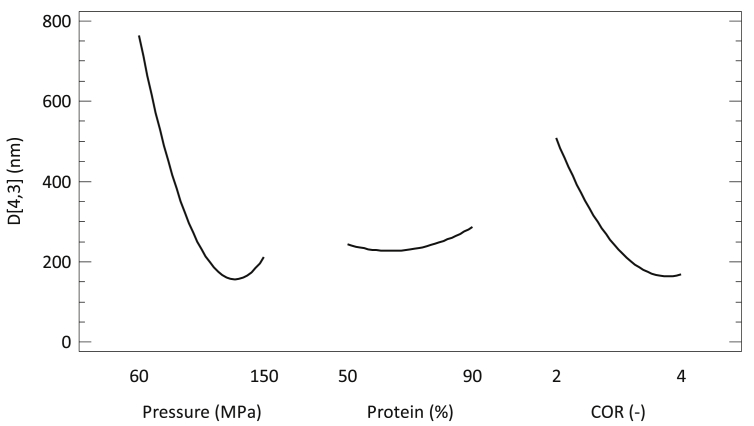
Main effects diagram for the emulsification process of rice bran oil.

The trend that the EDS has when changing the three process parameters studied in this work, is shown in the main effects diagram (Figure 2).

As can be seen, increases in pressure tend to reduce the average droplet diameter of the resulting emulsion to a minimum, but further increases in pressure tend to increase the droplet size. This is due to the presence of coalescence phenomena. COR also had statistically significant effect on the EDS, being necessary high ratios to decrease the droplet size. This means that there is a minimum amount of carrier needed to cover all the oil used in the emulsion. In the literature, it is possible to find that the most commonly used ratio is 4 (Gallardo et al., 2013; Melgosa et al., 2019; Moreno et al., 2016; Pereyra-Castro et al., 2018). By changing the COR to 2, given the fact that the solid content of the emulsion remains unaltered, the oil load in the emulsion is increased. In general, as reported Jafari et al. (2007a), higher oil contents in the emulsion make the EDS to increase, since the amount of carrier material available might be not enough to cover the newly formed droplets.

The composition of the carrier material did not have statistically significant effect on the droplet size of the emulsion, however an increase in the droplet size was detected the more pea protein was used. PPI is a complex mixture of high molecular weight proteins (mainly globulins (65–80%; they are a mixture of legumin (350–400 kDa), vicilin and convincilin (150 kDa)), albumins and glutelins (Nesterenko et al., 2013)), which makes it to be considered as slow emulsifier. In summary, big size and folded configuration make the proteins and other biopolymers to diffuse and adsorb onto the fresh interface much slower than conventional surfactants (Arboleya and Wilde, 2005), being the EDS reduction slow and gradual. It also has to be noted that the emulsification properties of pea protein are driven by their amphiphilic nature (Burger and Zhang, 2019); therefore, changes in the structure and configuration of the protein induced as a consequence of the processing in the interaction chamber can affect their functionality. Although the residence time in the interaction chamber is very low (10^−4^ s (Jafari et al., 2008b)), important effects on the protein structure happen: on the one hand there is a pure effect of the high pressure, that might induce conformational changes in the protein (Bouaouina et al., 2006); on the other hand, the pass through the interaction chamber produces cavitation, shear, turbulence and temperature rise.

**Figure 3:**
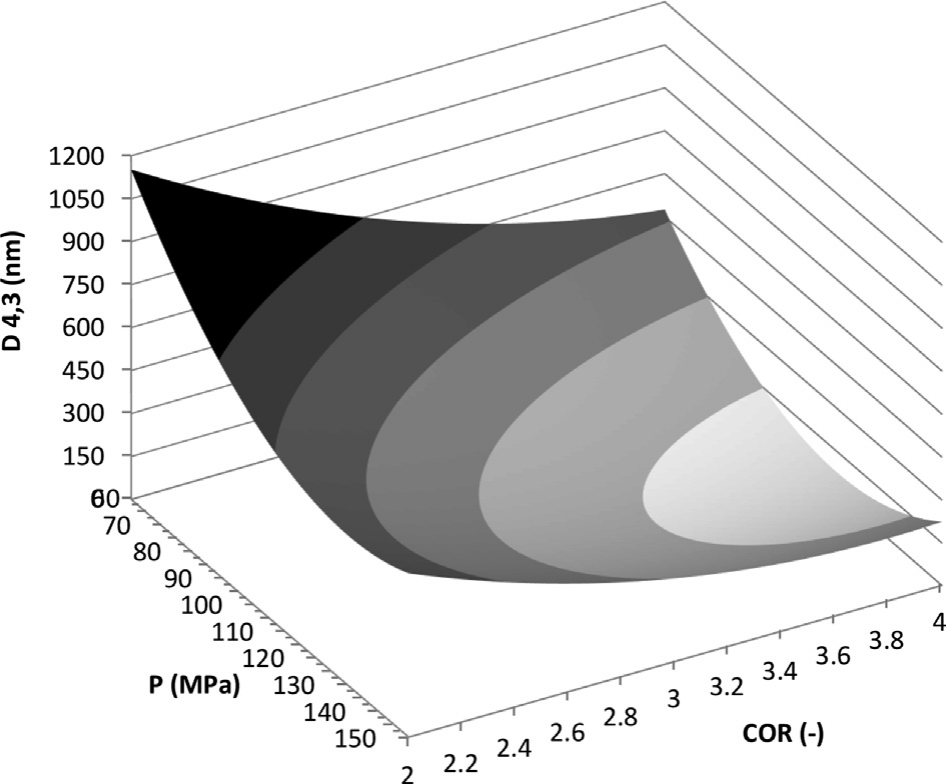
Surface diagram for the average droplet size of rice bran oil, using as carrier a mixture 70% PPI + 30% MD, as a function of the pressure and the COR. Key: , >750 nm; , 600–750 nm; , 450–600 nm; , 300–450 nm; , 150–300 nm; , < 150 nm.

The effect of the main process parameters can be seen in Figure 3, where a surface plot of the EDS changes with pressure and the COR at a fixed content of PPI in the carrier mixture is shown, and the trends described in the previous paragraphs can be observed.

#### 3.3.2 Interactions between experimental factors

According to the statistical analysis presented in Table 4 strong interactions between factors have been detected. There was strong interaction between pressure and carrier composition: at low homogenization pressures, the more protein in the carrier, the larger the EDS. However, at the highest working pressure, the opposite phenomenon was observed: the more protein in the carrier, the smaller the droplet size. At high pressure conditions the energy input is higher, so smaller droplets are formed and, in order to stabilize them, more protein is needed, since it seems that MD emulsification properties are not good enough.

**Figure 4:**
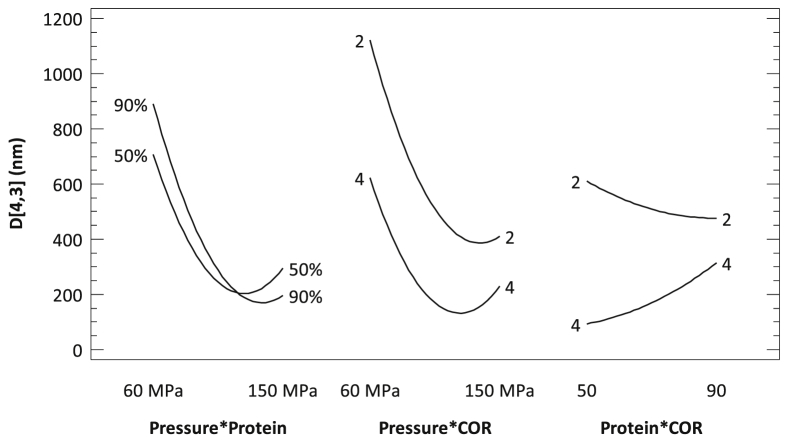
Interactions diagram for the emulsification process of rice bran oil.

There was also a strong interaction between pressure and COR. In general, as can be seen in Figure 4, the more carrier used (higher carrier to oil ratio) the lower EDS is achieved, regardless the pressure used. When COR was equal to 2, increases in the working pressure tended to reduce the particle size, and only at the highest working pressure range, an increase in the particle size was observed. Nevertheless, when the ratio used was equal to 4, increases in pressure reduced the droplet size (as expected, because there is more carrier material to cover the oil), but a fast increase in the average droplet size was observed when pressure was higher than 110 MPa (where the minimum droplet size was detected). It was under these high energy conditions when the EDS increased very fast. When COR = 4, there is more protein and the high pressures might be affecting the electrostatic balance or the tridimensional configuration of the protein, affecting its emulsifying properties.

The latest interaction between factors was detected between the composition of the carrier and COR. In this case at the lowest value of the COR (equal to 2, which means that only twice more carrier than oil is used in the emulsion), the increase in the amount of protein in the carrier mixture serves to decrease the average droplet size; all the protein present in the media is needed to cover and stabilize the new droplets formed, and therefore the EDS tends to decrease the more protein is present in the carrier. However, when COR equals to 4 (4 times more carrier than oil) increases in the protein content in the carrier material increase the average droplet size. Proteins are voluminous molecules and therefore, when COR is 4 and the 90% of the carrier is pea protein, is under those conditions that the highest amount of protein is being used to encapsulate the RBO. Probably there is an excess of protein that make coagulation-recoalesce phenomena likely to happen, resulting in an increase in the average droplet size.

Results presented by other authors confirm the complexity of the high pressure homogenization processes. Gumus et al. (2017) prepared emulsions with 10% oil, and vegetable proteins as carrier material in a concentration of 20 g/L, and passed 3 times though the microfluidizer at 69 MPa. The final D[3,2] of the resulting emulsions were around 376, 407, and 409 nm for faba bean, lentil, and pea proteins, respectively, values significantly higher than those obtained when whey protein isolate was used (130 nm). Ho et al. (2017) used dairy (whey and sodium caseinate) and plant (pea and soy) proteins to formulate lycopene in canola O/W emulsions. The coarse emulsion (10% of oil and 7 g/L pea protein dissolved in water) was passed through the microfluidizer for 5 times at 80 MPa. The sodium casseinate and PPI-stabilized emulsions exhibited similar particle size distributions at day 0 and day 14 (D[3,2] was around 0.18 μm). Santos et al. (Santos et al., 2018) used advanced performance xanthan gum to prepare egg protein and high oleic sunflower oil emulsions with one single pass through the microfluidizer at pressures up to 138 MPa, with a decrease in the EDS up to 10,000 psi; further increases in pressure resulted in an increase of the droplet size; Qian and McClements (2011) concluded than the minimum EDS was strongly dependent on the emulsifier type and concentration (sodium dodecyl sulfate < Tween 20<β-lactoglobulin < sodium caseinate) when working at 140 MPa and passing 6 times through the interaction chamber, in the case of Mazola Corn oil. These authors, who passed the emulsion up to 8 times through the chamber, observed a sharp decrease after the first pass, and then a constant decrease when increasing the number of passes. This observed decrease was faster the lower the pressure (the lower the pressure more passes are needed to reduce the EDS). According to Ho et al. (2017), PPI shows excellent emulsifying properties, related to the prevention of flocculation or coalescence of lycopene emulsions, results that were as good as those obtained when sodium casseinate was used.

As can be seen, the comparison of the experimental results among the authors is complex. It is possible to conclude that, in general, the emulsification process through microfluidization is very complex, since there are different factors affecting the process. Some of them are controllable (such as pressure and COR), but others are not easily controllable, especially those affecting the protein functionality (which is controlled by its composition and structure) such as the cultivar of the pea, the globulin fraction, the isolation method of the protein and the production scale (Burger and Zhang, 2019).

### 3.4 Optimization of the emulsification process

The statistical analysis of the experimental results allowed to conclude that the conditions that minimized the emulsion droplet size are pressure 114 MPa, carrier composition (64% pea protein isolate + 36% of maltodextrin) and carrier to oil ratio equal to 3.2. Under these experimental conditions, a confirmatory experiment (repeated tree times) was carried out. The experimental results obtained are presented in Table 5.

**Table 5:** Confirmatory experiment carried out at the optimal conditions that minimized the EDS.

Experimental conditions			Emulsion properties			
P (MPa)	PPI (%)	COR (-)	D[4,3] (nm) predicted	D[4,3] (nm) observed	D[3,2] (nm) observed	span (-) observed
114	64	3.2	174	189 ± 3	105 ± 1	1.52 ± 0.01

As can be seen, there was a correlation between the experimental results obtained and the results predicted by the model in the optimal conditions. The model tends to underestimate the EDS of the emulsion.

### 3.5 Emulsion stability

The stability of the emulsion obtained in the optimal conditions when stored at different temperatures (4 and 25 °C) was measured.

#### 3.5.1 Emulsion stability at 4 °C

**Figure 5:**
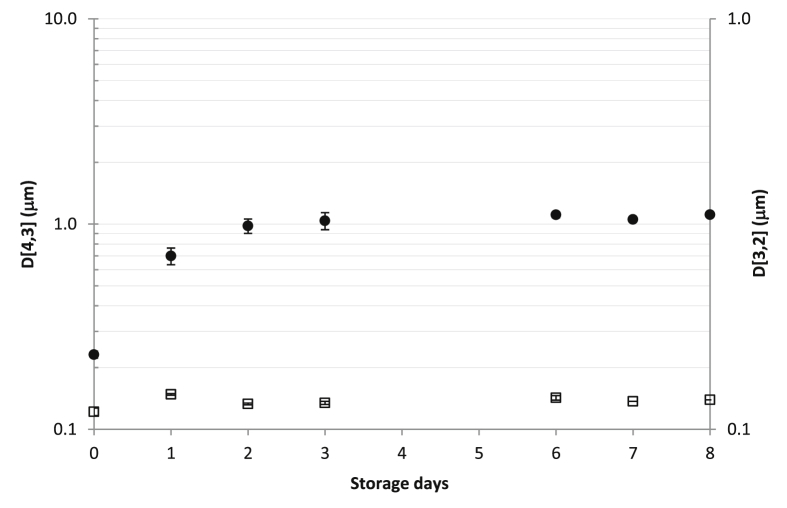
EDS (expressed as D[4,3] () and as D[3,2] ()) of the optimal emulsion stored at 4 °C.

As presented in Figure 5, the emulsion was rather stable when kept at 4 °C. There was an increase in the EDS up to 1 μm within the first two days, remaining unaltered for the subsequent 8 days. After the 8^th^ storage day, the EDS increased sharply and the coagulation of the emulsion was observed. Gumus et al. (2017) observed an important increase in the EDS after 33 days of storage at 37 °C. Samples prepared using lentil protein were monodisperse in day 0, but evolved after storage time to become a bimodal distribution, with an important increase of the big size particles.

In order to find out if the EDS increase was due to flocculation or coalescence, a deflocculant (such as SDS or Tween) could be added to the emulsion prior to the droplet size measurement, as described Santos et al. (2018). According to the data presented in Figure 5, after 8 days storage at 4 °C, D[4,3] of our sample was 1112 ± 23 nm; after the addition of SDS to the sample, the EDS was dramatically reduced to 286 ± 20 nm, indicating that the increase in the average droplet size was mainly due to flocculation, instead of coalescence. According to McClements (2015), when using biopolymers as emulsifiers, flocculation can occur due to the formation of bridges between two or more droplets. Hydrophobic and electrostatic interactions are the most common types of interactions in food emulsions, being both types of interactions able to form bridges between molecules.

The ζ-potential of the emulsion (kept at 4 °C) was also measured throughout 8 days. The initial value of this parameter was -32.5 ± 1.0 mV, a value that indicates a moderate stability of the emulsion. After that, ζ-potential tended to increase after 9 days at 4 °C: -31.1 ± 0.8 mV (day 4), -30.1 ± 0.8 mV (day 5 of storage) and -25.7 ± 0.4 mV(day 9). This increase in the ζ-potential is indicative of alterations in the interfacial composition of the protein-coated lipid droplets. Gumus et al. (2017) reported a ζ-potential around -21 mV for the emulsions prepared using pea protein.

#### 3.5.2 Emulsion stability at 25 °C

**Figure 6:**
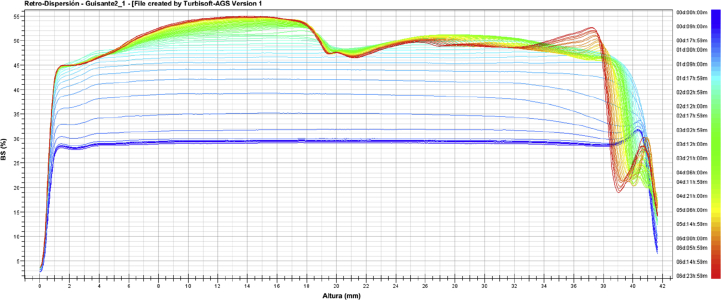
Changes in the backscattering profile (ΔBS) over time in the O/W emulsion.

The stability of the emulsion was measured at 25 °C in the AGS ageing station of the Turbiscan. The changes in the backscattering profile (ΔBS) of the O/W emulsion sample were recorded every 3 h for 7 days of storage at 25 °C and plotted vs. time. Results can be seen in Figure 6, where it is possible to see that the emulsion began to be unstable in less than 24 h. The explanation of the phenomenon observed seems to be difficult, as a complex migration is happening. First, in the central part of the sample an increase in the ΔBS is happening; this increase is uniform for the first 48 h. Then, it seems to be faster in the bottom of the sample (height up to 18 mm). In the present work, initial average droplet size of the optimal emulsion was around 0.2 μm. When particles are smaller than the wavelength of the incident light (880 nm in the Turbiscan), the Rayleigh diffusion is taking place. In this case diffusion is isotropic. With small particles (d < 0.6 μm), the backscattering flux increases with the diameter increase. It means that a particle size increase (flocculation or coalescence) of small particles will lead to an increase of the backscattering level, up to a critical value of the particles, when they get bigger than 1 mm. This is in agreement with the flocculation phenomena that had been observed in our samples, after the addition of the surfactant SDS, as presented in section 3.5.1.

**Figure 7:**
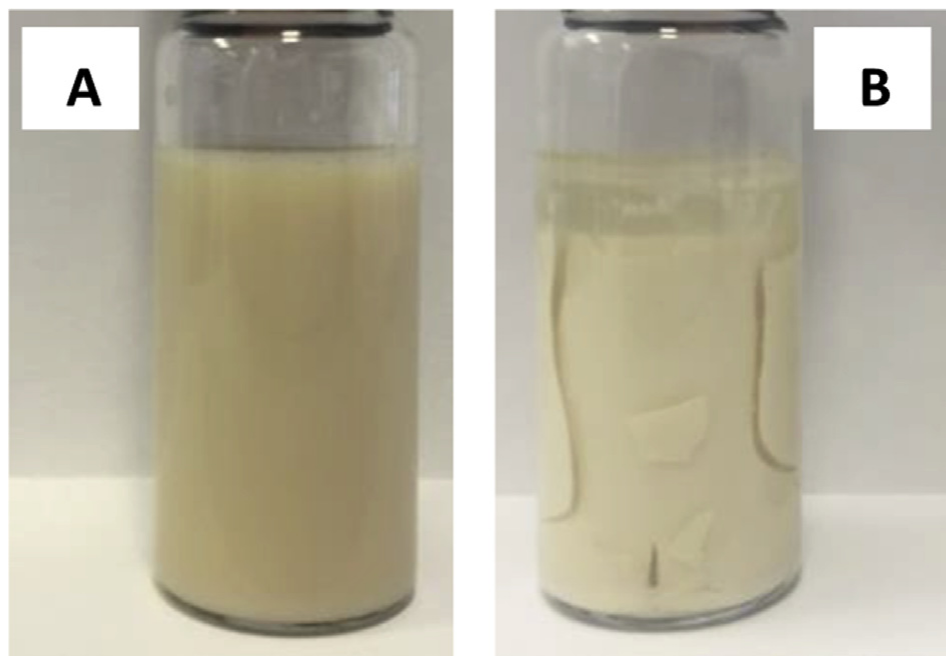
Initial emulsion (A) and emulsion after 7 days stored at 25 °C (B).

The second phenomenon that can be observed happened in the upper part of the sample (from 34 to 42 mm). After the first 24 h in which the value of ΔBS increased, it began to decrease. This is indicating a clarification of the sample. Particles tend to flocculate, providing a clear upper part of the sample. In Figure 7 theses two phenomena can be clearly observed: after 7 days at 25 °C the emulsion is completely coagulated and the clarification of the upper part is visible, in addition to a color change. This means that once the emulsion is prepared, it should be kept in cold or more preferably dried as a microencapsulated solid powder.

### 3.6 Emulsions drying. Characterization of the dried powders

In Table 6, the main characteristics of the particles obtained using different drying techniques are shown.

**Table 6:** Summary of drying experiments results.

Drying Technique	Yield (%)	EE (%)	Bioactive Loading (mg/g)	Moisture (%)	D[4,3] (μm)	D[3,2] (μm)	span
Spray Drying	50.8	74 ± 1^a^	173 ± 3^a^	2.6 ± 0.1^a^	18.8 ± 0.1^a^	11.9 ± 0.1^a^	1.8 ± 0.1^a^
PGSS Drying	59.9	53 ± 2^b^	124 ± 6^b^	2.2 ± 0.2^a^	11.6 ± 0.5^b^	6.2 ± 0.2^b^	3.0 ± 0.4^b^
Freeze Drying	96.9	96 ± 3^c^	226 ± 7^c^	2.8 ± 0.1^a^	-	-	-

∗Different letters in each column mean statistically significant differences at 95% confidence level.

#### 3.6.1 Yield and encapsulation efficiency

Spray drying and PGSS drying provided similar yields, in the range from 50 to 60%, common values for laboratory scale devices, whereas freeze drying yielded almost 100% of solid powder from the emulsion. All the powders obtained by the different drying technologies resulted to have very low residual moisture, below 3% in all cases.

Significantly bigger differences were observed regarding the encapsulation efficiency (EE). Particles obtained after the conventional spray-drying process had an encapsulation efficiency of 74 ± 1%, which was bigger than that obtained in the PGSS drying process (53 ± 2%). The encapsulation efficiency value was quite similar to other examples that can be found in the literature in which particles were obtained by spray drying: Murali et al. (2016) achieved an EE equal to 77% using tapioca starch and soy protein isolate as wall materials; Charoen et al. (Charoen et al., 2015) obtained higher encapsulation efficiencies (92–95%) when encapsulating rice bran oil (10% oil in the emulsion) using whey protein isolate (3.5%) and modified starch (7%) as wall material in a pilot scale spray dryer; Gupta et al. (Gupta et al., 2015) obtained lower EE (62.8%) when using modified starch Hi-Cap 100 as wall material; another example of rice bran oil encapsulation using spray drying was provided by Murali et al. (Murali et al., 2017), who reported and EE of 85.9% after using mixtures of whey protein and Jackfruit seed starch in a ratio 1:3; Pierucci et al. (Pierucci et al., 2007), when encapsulating α-tocopherol, found around 77.8% when using mixtures pea protein/maltodextrin (50/50), value that increased up to 86.8% when only pea protein was used as wall material. Results are difficult to compare among the authors due to the different wall materials used and the working conditions. The encapsulation efficiency of the particles obtained by PGSS drying, was significantly lower than other results presented in the literature. As can be seen in the literature, the EE is strongly dependent on the wall material and the working conditions: Melgosa el al (Melgosa et al., 2019). reported an EE close 100% in the case of encapsulation of fish oil in modified starch, or De Paz et al. (De Paz et al., 2012) who reported an EE not higher than 58.7% when encapsulating β-carotene in soy lecithin (liposomes).

Related to the EE, the bioactive loading, resulted to be 173 ± 3 mg oil/g sample for the spray-drying process, a reasonably good value considering the theoretical value of 235 mg/g sample. The bioactive loading of the particles obtained using PGSS drying was 28% lower (124 ± 6 mg oil/g sample). For the calculations it has been considered only the encapsulated oil, not the free oil.

#### 3.6.2 Particle size analysis

**Figure 8:**
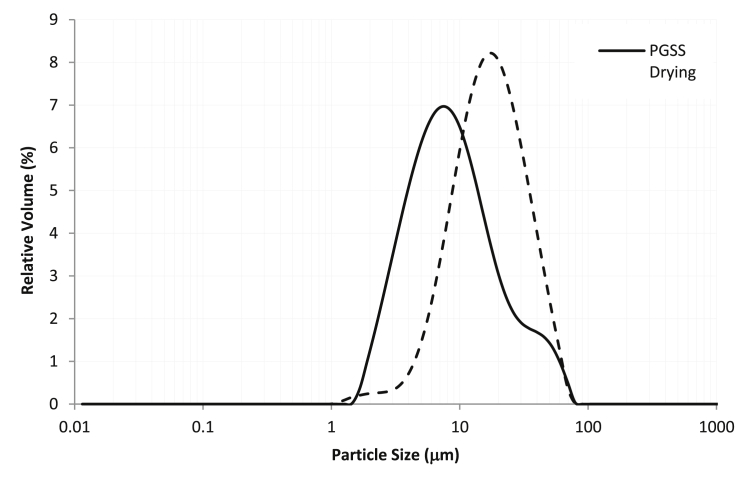
Particle size distribution plot of the particles obtained by PGSS-drying and by spray-drying.

The particle size distribution for PGSS-dried and spray-dried particles is plotted in Figure 8. Both distributions tended to be monodisperse, but differences were observed. The conventional spray drying yielded particles with an EDS equal to 18.8 ± 0.1 μm, whereas PGSS-dried particles had and EDS lower (11.6 ± 0.5 μm). As it has been reported in previous works (De Paz et al., 2012; Melgosa et al., 2019) the effective atomization caused by CO_2_ vaporization may be responsible for the formation of smaller and monodisperse distributions.

The span value of the PGSS-dried particles (3.0) was higher than that of the spray-dried particles (1.8), due to the presence of a small shoulder in the larger particle size zone, which is probably indicating the agglomeration of particles due to the presence of non-encapsulated oil. The analysis of the SEM images will be useful to shed light on the morphology of the particles and the presence of clusters.

The stability of the particles was checked after two weeks of storage at 4 °C. The EE decreased in both the particles obtained by spray drying and PGSS drying to 66 ± 1% and 49 ± 2%, respectively. In the case of the particles obtained by freeze drying no changes were observed. These changes in the EE were also observed in the average size of the particles obtained by spray and PGSS drying. In both cases particle size increased up to 25.6 ± 0.1 μm and 18.4 ± 1.2 μm, respectively.

#### 3.6.3 Particle morphology (SEM)

**Figure 9:**
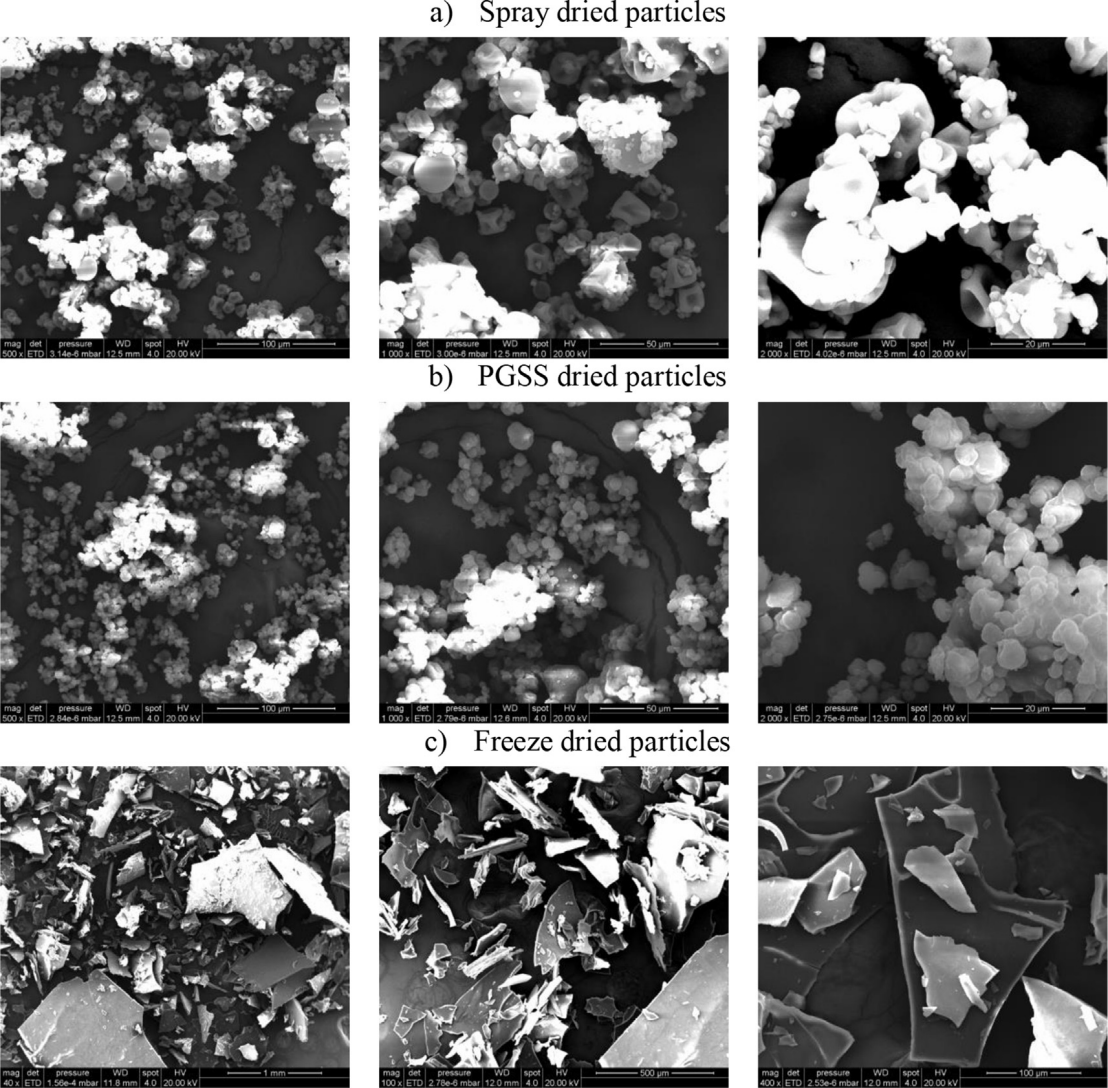
SEM micrographs of the dried powders using different drying techniques: spray drying (a); PGSS drying (b) and freeze drying (c). Magnifications are 500, 1000, 2000x (from left to right) for the spray and PGSS dried powders, whereas in the case of the freeze dried powders are 40, 100 and 400x (from left to right).

The morphology of the powders obtained using three different drying technologies (spray drying, PGSS drying and freeze drying) are shown in Figure 9. It is possible to see the typical appearance for the spray dried particles: they are shrunk and dented. This appearance has been reported by several authors using different wall materials, such as modified starch (Melgosa et al., 2019), pea protein (Moreno et al., 2016), different biopolymers (Gallardo et al., 2013), or mixtures of maltodextrin and soy protein isolate (Carneiro et al., 2013), and it is probably due to the fast evaporation of water, that results in the collapse of the particles during the drying process. It is also possible to see small droplets attached to the surface of the particles, which is likely to be the oil released from the core of the particle, which induces the formation of clusters. It is also possible to see differences in the size of the spray-dried particles.

Compared to the spray-dried particles, particles obtained using PGSS drying resulted to be more spherical and regular in terms of shape; however big clusters tended to appear, probably due to the lower encapsulation efficiency that induces the aggregation of the particles. In the particle size distribution (Figure 8) a shoulder at larger particle size was observed, something that has been also observed in the SEM images. To the best of our knowledge this is the first time SEM images of pea protein particles obtained by PGSS drying are reported.

In the case of the freeze-dried particles, these had a flake-like appearance, with sizes larger than 100 μm (Figure 9c). In general larger and more irregular particles have been produced when using this technique.

## 4 Conclusions

The encapsulation of rice bran oil using pea protein and maltodextrin mixtures as wall material resulted to be complex and affected by several operational parameters, which exhibited strong interactions among them. Although the emulsions obtained in the optimal emulsification conditions can have droplet diameters below 200 nm, these emulsions stabilized by pea protein are unstable even when stored at 4 °C, due to the appearance of flocculation phenomena. This fact recommended the emulsions drying right after being obtained. The final appearance of the powder depended on the drying technique: spray drying provided higher encapsulation efficiencies than PGSS drying. In both cases powder particle size was below 20 μm, but PGSS-dried emulsions yielded powders with spherical particles whereas spray-dried particles were shrunk and dented. Freeze-dried emulsions yielded the highest encapsulation efficiency and the highest oil retention along the time.

## Declarations

### Author contribution statement

Óscar Benito-Román: Conceived and designed the experiments; Performed the experiments; Analyzed and interpreted the data; Contributed reagents, materials, analysis tools or data; Wrote the paper.

María T. Sanz: Analyzed and interpreted the data; Contributed reagents, materials, analysis tools or data; Wrote the paper.

Sagrario Beltrán: Analyzed and interpreted the data; Wrote the paper.

### Funding statement

This work and O.Benito-Román's Post-doctoral contract were supported by Junta de Castilla y León and the European Regional Development Fund (ERDF) (Project BU301P18).

### Competing interest statement

The authors declare no conflict of interest.

### Additional information

No additional information is available for this paper.
